# Location-Dependent Patient Outcome and Recurrence Patterns in IDH1-Wildtype Glioblastoma

**DOI:** 10.3390/cancers11010122

**Published:** 2019-01-21

**Authors:** Christine Jungk, Rolf Warta, Andreas Mock, Sara Friauf, Bettina Hug, David Capper, Amir Abdollahi, Jürgen Debus, Martin Bendszus, Andreas von Deimling, Andreas Unterberg, Christel Herold-Mende

**Affiliations:** 1 Division of Experimental Neurosurgery, Department of Neurosurgery, University Hospital Heidelberg, INF 400, D-69120 Heidelberg, Germany; rolf.warta@med.uni-heidelberg.de (R.W.); andreas.mock@med.uni-heidelberg.de (A.M.); sara.friauf@med.uni-heidelberg.de (S.F.); andreas.unterberg@med.uni-heidelberg.de (A.U.); h.mende@med.uni-heidelberg.de (C.H.-M.); 2 Department of Medical Oncology, National Center for Tumor Diseases, INF 460, D-69120 Heidelberg, Germany; 3 Department of Neuroradiology, Clinic of Neurology, University Hospital Heidelberg, INF 400, D-69120 Heidelberg, Germany; bettina.hug@muerle.de (B.H.); martin.bendszus@med.uni-heidelberg.de (M.B.); 4 Department of Neuropathology, Institute of Pathology, University of Heidelberg, German Cancer Consortium, CCU Neuropathology, German Cancer Research Center, INF 224, D-69120 Heidelberg, Germany; david.capper@charite.de (D.C.); andreas.vondeimling@med.uni-heidelberg.de (A.v.D.); 5 Department of Neuropathology, Charité-Universitätsmedizin, Charitéplatz 1, D-10117 Berlin, Germany; 6 Department of Radiation Oncology, Heidelberg University Hospital, Molecular and Translational Radiation Oncology, National Center for Tumor Diseases, German Cancer Research Center, D-69120 Heidelberg, Germany; amir.abdollahi@med.uni-heidelberg.de (A.A.); juergen.debus@med.uni-heidelberg.de (J.D.)

**Keywords:** Glioblastoma, isocitrate dehydrogenase 1 (IDH1)-wildtype, subventricular zone, survival, multifocal growth, distant recurrence

## Abstract

Recent studies suggest that glioblastomas (GBMs) contacting the subventricular zone (SVZ) as the main adult neurogenic niche confer a dismal prognosis but disregard the unique molecular and prognostic phenotype associated with isocitrate dehydrogenase 1 (IDH1) mutations. We therefore examined location-dependent prognostic factors, growth, and recurrence patterns in a consecutive cohort of 285 IDH1-wildtype GBMs. Based on pre-operative contrast-enhanced MRI, patients were allotted to four location-dependent groups with (SVZ+; groups I, II) and without (SVZ−; groups III, IV) SVZ involvement or with (cortex+; groups I, III) and without (cortex−; groups II, IV) cortical involvement and compared for demographic, treatment, imaging, and survival data at first diagnosis and recurrence. SVZ involvement was associated with lower Karnofsky performance score (*p* < 0.001), lower frequency of complete resections at first diagnosis (*p* < 0.0001), and lower non-surgical treatment intensity at recurrence (*p* < 0.001). Multivariate survival analysis employing a Cox proportional hazards model identified SVZ involvement as an independent prognosticator of inferior overall survival (*p* < 0.001) and survival after relapse (*p* = 0.041). In contrast, multifocal growth at first diagnosis (*p* = 0.031) and recurrence (*p* < 0.001), as well as distant recurrences (*p* < 0.0001), was more frequent in cortex+ GBMs. These findings offer the prospect for location-tailored prognostication and treatment based on factors assessable on pre-operative MRI.

## 1. Introduction

Glioblastoma (GBM) World Health Organization (WHO) grade IV, the most common and lethal primary brain tumor, is a clinically, radiographically, and molecularly heterogeneous disease. Standard therapy comprising maximal safe resection, irradiation, and temozolomide (TMZ)-based chemotherapy confers a median survival of less than 15 months [1], with high inter-individual variability and only 3–5% of patients experiencing long-term survival (LTS) of more than three years [2]. Understanding the ontogeny of GBM, and mechanisms leading to its heterogeneity, will help to tailor individualized treatments based on personalized prognostication and improve patient outcomes. 

Mounting evidence suggests that glioma ontogeny is linked to a subpopulation of neural stem (NSC) and progenitor (NPC) cells persisting in neurogenic niches throughout adulthood, in particular NSC-like astrocytes in the subventricular zone (SVZ) lining the walls of the lateral ventricles [3,4]. In animal models, inactivation of tumor suppressor genes (TP53, NF1, PTEN) or activation of oncogenes (Akt, Ras) resulted in the formation of astrocytomas through malignant transformation of NSCs [5,6,7,8]. In humans, the SVZ was identified as a reservoir of malignant precursor clones by employing a novel approach of intraoperative fluorescence-guided multiple sampling of GBMs and their adjacent fluorescent SVZ [9]. Accordingly, targeting the ipsilateral SVZ by postoperative irradiation has been shown to confer improved progression-free (PFS) and overall (OS) survival [10]. Ultimately, several studies have reported that the proximity of GBM to the SVZ is associated with inferior patient outcomes [11,12,13,14,15,16]. However, none of these studies considered isocitrate dehydrogenase 1 (IDH1) mutation status for survival analysis, although IDH1-mutant (IDH1-mut) gliomas comprise a molecularly distinct GBM subgroup with favorable patient outcomes [17,18]. This has prompted the 2016 WHO classification of CNS tumors to discriminate between IDH1-wildtype (IDH1-wt) and IDH1-mut GBMs [19]. Moreover, conflicting data exist regarding the impact of SVZ involvement on growth and recurrence patterns of GBM. Initially, Lim et al. reported a series of 53 GBMs assigned to four groups dependent on their spatial relationship to the SVZ and the cortex [20]. Group I GBMs (contacting SVZ and cortex) were most frequently multifocal at the first diagnosis and recurred in a remote location, while group IV GBMs (neither contacting SVZ nor cortex) were always solitary lesions with tumor recurrence exclusively adjacent to the resection cavity [20]. Accordingly, Adeberg et al. reported that distant and multifocal progression was more common in GBMs contacting the SVZ [21]. In contrast, Kappadakunnel et al. found the highest rate of multifocal disease in group III GBMs (involving cortex but not SVZ), while the presence of distant tumor recurrence was independent of tumor location [12]. Intriguingly, in a MRI analysis of 49 GBM patients, no location-dependent recurrence pattern was observed [22]. Finally, computerized simulation of glioma growth provided evidence that GBMs involving the SVZ do not necessarily originate in the periventricular region, but also in the white matter with centrifugal growth and ultimate contact to the SVZ, dependent on increasing tumor size [23]. To shed light into these conflicting data, possibly owing to small sample sizes and the inherent molecular bias conferred by IDH1 mutations, we sought to analyze a large cohort of 285 patients with newly diagnosed IDH1-wt GBM with respect to location-dependent survival, growth, and recurrence patterns. 

## 2. Results

### 2.1. Location-Dependent IDH1 Mutation Status and Treatment-Inherent Differences

**Figure 1:**
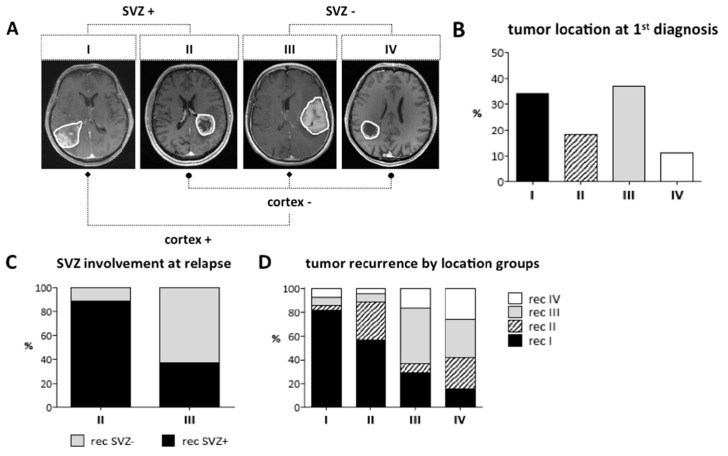
As exemplified in (**A**), 285 IDH1-wt GBM patients were allocated to four different location groups based on the CEL’s contact to the SVZ and/or the cortex on pre-operative MRI. Groups were further pooled into GBMs with (SVZ+) and without (SVZ−) SVZ involvement or with (cortex+) and without (cortex−) cortical involvement. (**B**) At 1st diagnosis, most GBMs were allocated to groups III (37%) and I (34%). (**C**) SVZ involvement increased from 1st diagnosis to recurrence, as depicted for “pure” SVZ (group II) and “pure” cortical (group III) GBMs. Group III tumors now recurred as SVZ+ GBM in 38.5% of cases while only 11% of group II tumors shed their SVZ contact and recurred as SVZ− GBM. (**D**) No predilection was found for each of the four groups regarding location of recurrent tumor. Most group I and III GBMs recurred at the same location, while recurrences of group IV GBMs were distributed throughout all groups.

Altogether, 302 consecutive newly diagnosed GBM patients were allocated to one of the four location groups depicted in Figure 1A. Group I consisted of contrast-enhancing lesions (CEL) contacting the SVZ and infiltrating the cortex, group II of CELs contacting the SVZ only, group III of CELs contacting the cortex only, and group IV of CELs residing in the subcortical white matter, neither contacting SVZ nor cortex. Location-dependent molecular and clinical aspects, growth and recurrence patterns and patient outcome were investigated in each of the following comparisons: SVZ+ (groups I, II) vs. SVZ− (groups III, IV); cortex+ (groups I, III) vs. cortex− (groups II, IV); and group II (“pure” SVZ involvement) vs. group III (“pure” cortical involvement). IDH1 mutations were identified in 17/302 patients (5.6%), leaving 285 IDH1-wt patients for further comparison. Noteworthy, IDH1-mut tumors were more common among GBMs without SVZ involvement (SVZ+ vs. SVZ−: *p* = 0.04). Demographic, radiographic, treatment-related and outcome data of the IDH1-wt GBM cohort are summarized in Table 1. Radiographic classification assigned the majority of tumors to group III (cortical involvement only; 37%) and group I (SVZ and cortical involvement; 34%) with a balanced distribution between SVZ+ (52%) and SVZ− (48%) patients (Table 1, Figure 1B). No location-dependent age difference was observed. Karnofsky performance score (KPS) was significantly lower in patients with SVZ involvement (SVZ+ vs. SVZ−: *p* < 0.001; II vs. III: *p* = 0.002). At first diagnosis, gross total resection (GTR) was achieved significantly more often in patients without SVZ involvement (group III: 47%; group IV: 45%; SVZ+ vs. SVZ−: *p* < 0.0001; II vs. III: *p* = 0.003), while intensified postoperative treatment was independent of tumor location. At relapse, MRI was available in 187/285 patients (66%) of whom 163 patients (87%) received any kind of salvage therapy. Neither the number of re-resections, nor the extent of resection (EOR), showed location-dependent differences; however, the number of non-surgical interventions (“non-surgical treatment intensity”) was significantly higher among SVZ− GBM patients (*p* < 0.001).

### 2.2. Location-Dependent Growth and Recurrence

**Figure 2:**
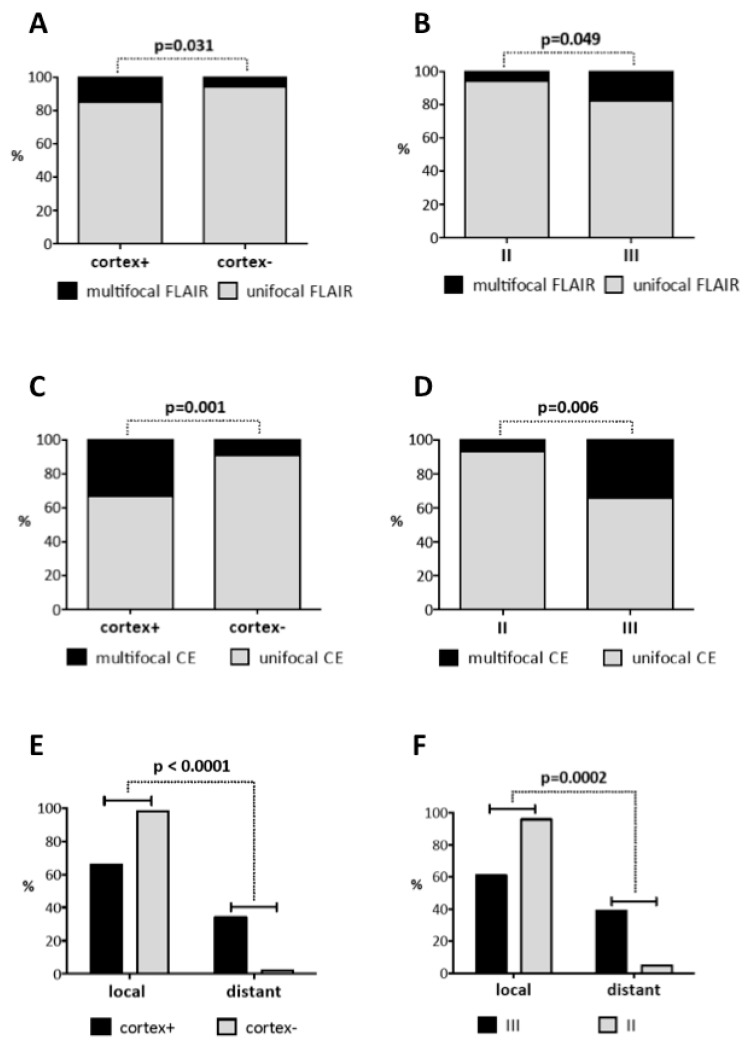
Multifocal growth and distant recurrences were hallmarks of cortical IDH1-wt GBMs. At 1st diagnosis (*n* = 285), multifocal FLAIR lesions were significantly increased in cortex+ vs. cortex− (**A**) and group III vs. group II GBMs (**B**), but not in SVZ+ vs. SVZ− GBMs. At recurrence (*n* = 187), multifocal CELs were significantly enhanced in cortex+ (**C**) and group III GBMs (**D**). Local and distant recurrence patterns differed markedly within location-specific groups. Distant tumor growth remote from the initial resection site was significantly more frequent in GBMs with cortical involvement ((**E**) cortex+ vs. cortex−; (**F**) group II vs. group III).

Next, conflicting data in the recent literature on location-dependent GBM growth and recurrence patterns prompted us to investigate these features in our large, molecularly homogeneous dataset. Multifocal disease noncontiguous with the primary CEL was evaluated both on contrast-enhancing (CE) T1-weighted (T1-w) and fluid-attenuated inversion recovery (FLAIR) images. At first diagnosis, multifocal disease was detected mainly in GBMs with cortical involvement (group III: 17% (CE), 18% (FLAIR); group I: 15% (CE), 13% (FLAIR)) with a significant increase in multifocal FLAIR lesions in cortex+ vs. cortex− (*p* = 0.031) or group III vs. II GBMs (*p* = 0.049), but not in SVZ+ vs. SVZ− GBMs (Figure 2A,B). Longitudinal analysis of all patients with MRI available at tumor relapse (*n* = 187) revealed an increase in patients with multifocal disease compared to the first diagnosis, again with a significant difference in cortex+ vs. cortex− (CE: *p* = 0.001; FLAIR: *p* = 0.164) or group III vs. II GBMs (CE: *p* = 0.006; FLAIR: *p* = 0.096), but this time particularly in CELs (group III: 34% (CE), 22% (FLAIR); group I: 32% (CE), 12% (FLAIR)) (Figure 2C,D). Thus, multifocal disease was primarily found in cortical GBMs and increased with tumor relapse. 

While most GBMs were evenly allocated to groups III (37%) and I (34%) at first diagnosis, group I GBMs represented the majority of tumors at relapse (48%; Table 1). Accordingly, SVZ involvement was increased to 61% of all recurrent GBMs compared to 52% at first diagnosis (Table 1). When comparing the two most unambiguous location groups II (SVZ involvement only) and III (cortical involvement only), group III GBMs now were found to extend towards the SVZ and recur as SVZ+ GBMs in 38.5% of cases while only 11% of group II GBMs shed their SVZ contact and recurred as SVZ− GBMs (Figure 1C). No predilection was found for each of the four groups regarding the location of recurrent tumor, although most group I and III GBMs recurred at the same location (group I: 82% rec group I; group III: 46% rec group III; Table 1, Figure 1D). In contrast, recurrences of group IV GBMs (neither SVZ nor cortical involvement) were observed throughout all groups (Table 1, Figure 1D). Of 187 patients with recurrent GBM, the vast majority (94%; *n* = 176) presented with local tumor recurrence adjacent to the primary resection site, among those 38 patients (20%) with concurrent distant tumor growth. Tumor growth exclusively remote from the primary resection site occurred in only 11 patients (6%) (Table 1). Local and distant recurrence patterns differed markedly within location-specific groups (*p* = 0.0001). Distant tumor growth was most commonly observed in group III (30%) and I (28%) GBMs resulting in a significant increase in distant tumor recurrences in GBMs with cortical involvement (cortex+ vs. cortex−: *p* < 0.0001; II vs. III: *p* = 0.0002) (Figure 2E,F).

In summary, tumor location had a distinct impact on tumor growth and recurrence: GBMs with cortical involvement were prone to grow at multifocal sites and to recur distant from the primary resection site.

### 2.3. Location-Dependent Patient Outcome

**Figure 3:**
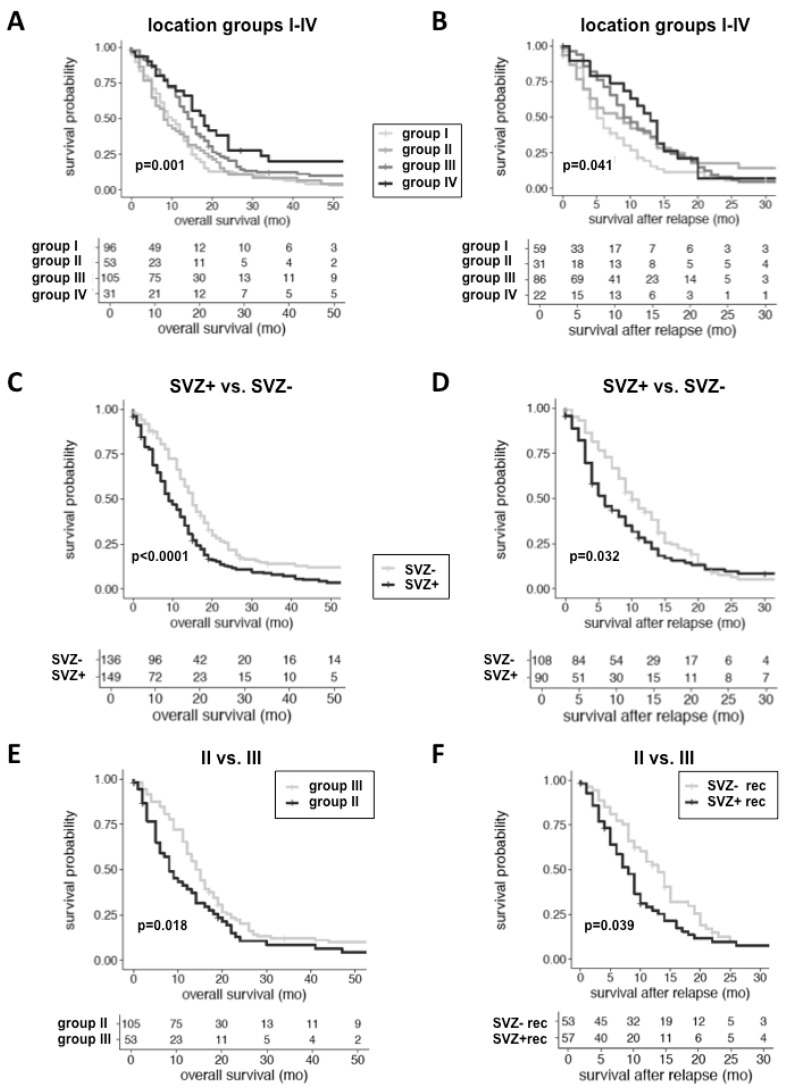
Kaplan-Meier plots depicting the negative prognostic impact of SVZ involvement in 285 IDH1-wt GBM patients; numbers at risk are given. OS and survival after relapse, but not PFS differed significantly when comparing all location groups (**A**,**B**), SVZ+ vs. SVZ− GBMs (**C**,**D**) and group II vs. group III GBMs (*n* = 155) (**E**,**F**). Shortest median OS and survival after relapse were observed in group I (10 and 5 months) and group II (8 and 6 months) patients. For comparison of survival after relapse in group II vs. group III GBMs, SVZ involvement at recurrence (SVZ+/− rec) was valued.

Previous studies reported that GBMs with SVZ involvement confer inferior survival [11,12,13,14,15,16] but disregarded the IDH1 mutation status as a major prognostic confounder. Since we found IDH1-mut tumors to be more frequent among SVZ− GBMs, we analyzed location-dependent survival exclusively in IDH1-wt GBMs. To this end, we compared SVZ+ vs. SVZ− GBMs as well as group II vs. III GBMs, the latter being the most unambiguous separation between GBMs with SVZ and cortical involvement. In univariate analysis of the complete IDH1-wt cohort (*n* = 285), both OS and survival after relapse differed significantly with longest median survival observed in group IV and shortest median survival in group II patients (OS: *p* < 0.001; survival after relapse: *p* = 0.041 (Table 1, Table S2, Figure 3A,B). Noteworthy, PFS was comparable between groups (*p* = 0.197). In general, SVZ involvement conferred inferior OS (*p* < 0.0001) and survival after relapse (*p* = 0.023) (Figure 3C,D). There was no location-dependent preponderance of STS or LTS (Table 1). Since cortical GBMs showed a tendency to extend towards the SVZ upon tumor relapse, we included SVZ involvement at recurrence (SVZ+ rec) into our prognostic model and found this also to be negatively associated with OS and survival after relapse (OS: *p* < 0.001; survival after relapse: *p* < 0.001; Table S2). Beside tumor location, well-known demographic and clinical parameters were identified as prognostic factors for OS (age at 1st diagnosis, pre-operative KPS, GTR and intensified treatment at the first diagnosis and recurrence), PFS (age, GTR and intensified treatment at the first diagnosis), and survival after relapse (age at the first diagnosis; GTR and intensified treatment at 1st diagnosis and recurrence) (Table S2). Interestingly, multifocal disease at the first diagnosis also predicted inferior OS (CEL: *p* = 0.003; FLAIR lesions: *p* = 0.028), PFS (CEL: *p* = 0.034; FLAIR lesions: *p* = 0.002) and survival after relapse (CEL: *p* = 0.005) (Table S2). The important prognostic impact of SVZ involvement was confirmed by multivariate analysis in which SVZ involvement at the first diagnosis was identified as an independent prognostic factor of inferior OS (*p* = 0.008) and SVZ involvement at the recurrence of inferior survival after relapse (*p* = 0.015) (Table 2).

Previous outcome studies analyzed group I and II tumors together. However, group I GBMs are voluminous tumors extending from the SVZ throughout the white matter to the cortex and may as well originate from the cortex spreading towards the SVZ. Consequently, we performed a survival analysis, exclusively comparing group II (SVZ involvement only) and III (cortical involvement only) patients. In line with our previous findings, OS was significantly shorter for “pure” SVZ patients (*p* = 0.028) (Figure 3E). Likewise, SVZ involvement at recurrence was associated with inferior survival after relapse (*p* = 0.045; Table S3, Figure 3F). Consistent with the complete cohort, age, EOR, intensified treatment as well as multifocal disease on CE and FLAIR images at 1st diagnosis were predictive of OS and PFS, while EOR and intensified treatment at recurrence were predictive of survival after relapse (Table S3). In multivariate analysis, SVZ involvement was confirmed as an independent prognostic factor for OS (*p* = 0.007), while tumor location no longer impacted survival after relapse (Table 3). 

In conclusion, SVZ involvement both at 1st diagnosis and recurrence needs to be considered as an important prognostic factor for OS and survival after relapse in IDH1-wt GBM.

## 3. Discussion

GBM is characterized by high molecular heterogeneity affecting clinical and radiographic presentation, treatment response, and survival. The underlying mechanisms are still poorly understood. Therefore, we interrogated if tumor location, particularly proximity to the SVZ, contributes to heterogeneous growth, recurrence patterns and patient outcome [16,20]. It has consistently been shown that IDH1 mutations entail a molecularly and prognostically distinct GBM subtype [17,18]. Interestingly, in this study, IDH1-mut tumors were significantly enriched in SVZ− GBMs. This is in line with an immunohistochemical study that detected IDH1 mutations more frequently among group III GBMs, admitted that newly diagnosed and secondary GBMs were analyzed together [24]. Our finding contributes to the hypothesis of location-specific molecular signatures, but also stresses the need to stratify any location-dependent survival analysis for IDH1 mutation status. To eliminate this molecular bias, we only analyzed IDH1-wt GBM patients (*n* = 285) and found remarkable location-specific differences. First, growth and recurrence patterns were dependent on cortical involvement. Multifocal growth, both at first diagnosis (FLAIR lesions) and recurrence (CEL), was significantly enhanced in cortex+ and group III GBMs. Additionally, distant recurrences were observed more often in these tumors. While SVZ involvement was increased from newly diagnosed to recurrent tumors, it did not affect growth and recurrence patterns. In contrast, SVZ involvement at the first diagnosis and recurrence was found to be an independent prognostic factor for inferior OS (SVZ+ GBMs, group II GBMs) and survival after relapse (SVZ+ GBMs), while cortical involvement did not impact on survival.

Our finding that cortical involvement predicts tumor growth and recurrence resolves the ambiguity of previous studies. Lim et al. reported that group I tumors were most often multifocal at first diagnosis [20], whereas Kappadakunnel et al. found the highest rate of multifocal disease, both at first diagnosis and recurrence, in group III tumors [12]. This might account to small sample sizes but also to the fact that Kappadakunnel et al. [12] only analyzed CELs, while Lim et al. [20] considered, but did not discriminate for, CE and FLAIR lesions. In our study, multifocal growth was comparably high in group I and III GBMs (CE: 15% and 17%; FLAIR: 13% and 18%) in contrast to only 6% of group II and IV GBMs and supports the data by Kappadakunnel et al. [12]. Indeed, although Lim et al. concluded that multifocality is characteristic of SVZ involvement, they encountered multifocal growth more often in tumors with (group I: 56%, group III: 29%) than without (group II: 11%, group IV: 0%) cortical involvement. In this context, it is impossible to compare the finding by Adeberg et al. that multifocal progression was more common in SVZ+ GBMs since this study discriminated only by SVZ involvement, but not by location-specific groups [21]. Noteworthy, group I tumors extend from the SVZ to the cortex and therefore can be assigned both to SVZ+ and cortex+ GBMs although their true spatial origin remains unknown. In fact, a computerized simulation model of GBM growth suggested that a tumor invading both cortex and SVZ more likely originates from the subcortical white matter than from the SVZ and that SVZ involvement is rather a matter of increasing tumor size [23]. This is in line with our own findings that group I GBMs represented the majority of tumors at relapse as opposed to group III GBMs at first diagnosis and that SVZ involvement increased from first diagnosis to recurrence. To overcome this potential bias for our radiographic and survival analysis, “pure” cortical (group III) and “pure” SVZ (group II) tumors were compared separately, still analyzing a significant number of patients (*n* = 158). Consistent with the complete cohort, we found a significant increase of multifocal lesions in “pure” cortical GBMs. The incidence of multifocal lesions in GBM is reported to range between 10–15% [25], but can be as high as 35% [26,27], and is considered exceedingly aggressive with significantly worse outcome compared to unifocal lesions [25]. This is supported by our multivariate survival analysis in which multifocal disease at first diagnosis independently predicted shorter PFS and OS (complete cohort), while multifocal disease at tumor relapse predicted shorter survival after relapse (group II vs. III). There is still uncertainty whether multifocal lesions arise from the same precursor cell. Two recent publications applying extensive genomic analyses obtained conflicting results, either describing that multiple lesions are derived from different clones with heterogeneous drug response [28] or reporting that multiple lesions are of monoclonal origin and share an unexpected high frequency of genetic alterations in core regulatory pathways (RTK/PI3K, p53, RB), which may account for their highly invasive phenotype [29]. In this regard, our finding that multifocal growth is location-dependent may indicate that these tumors are derived from different location-specific cells of origin with varying migratory potential.

GBM is characterized by its inevitable recurrence. In more than 80 %, tumors recur adjacent to the initial resection site [30,31], as confirmed by our radiographic analysis. In 20% of all cases, tumors recurred both locally and remote from the initial resection site while exclusive distant tumor recurrence was infrequent (6%). However, our analysis adds a remarkable location-specific picture since distant tumor recurrence was more frequently encountered in GBMs with cortical involvement (cortex+ GBMs, group III GBMs). In this respect, previous location studies reported ambiguous findings: Two small-sized studies did not observe any location-dependent recurrence pattern [12,22], while others reported that distant recurrence was more common in GBMs with SVZ involvement, but did not discriminate between all four location-specific groups [21]. Thus far, O^6^-methylguanin-DNA-methyltransferase (MGMT) promoter methylation [32], extensive resections [33], and large tumor volumes [34] were identified as risk factors for distant tumor recurrence. Our study identified cortical involvement (i.e., groups I (the largest tumors) and III (the highest frequency of GTR)) as another risk factor. From a molecular point of view, distant recurrence may reflect a highly invasive phenotype attributed to a distinct (possibly location-dependent) cell of origin, but experimental evidence is sparse. A recent longitudinal analysis found distant GBM recurrences to have a low rate of retention of the primary tumor’s driver mutations, indicating a divergent, rather than a clonal, evolution [35]. This finding is critical for guiding targeted therapies at relapse and advocates repeat surgery, particularly in distant recurrences. For prevention of tumor relapse, it is worth elucidating molecular drivers of distant recurrences for which cortical GBMs, based on our findings, seem to be the ideal workhorse.

**Figure 4:**
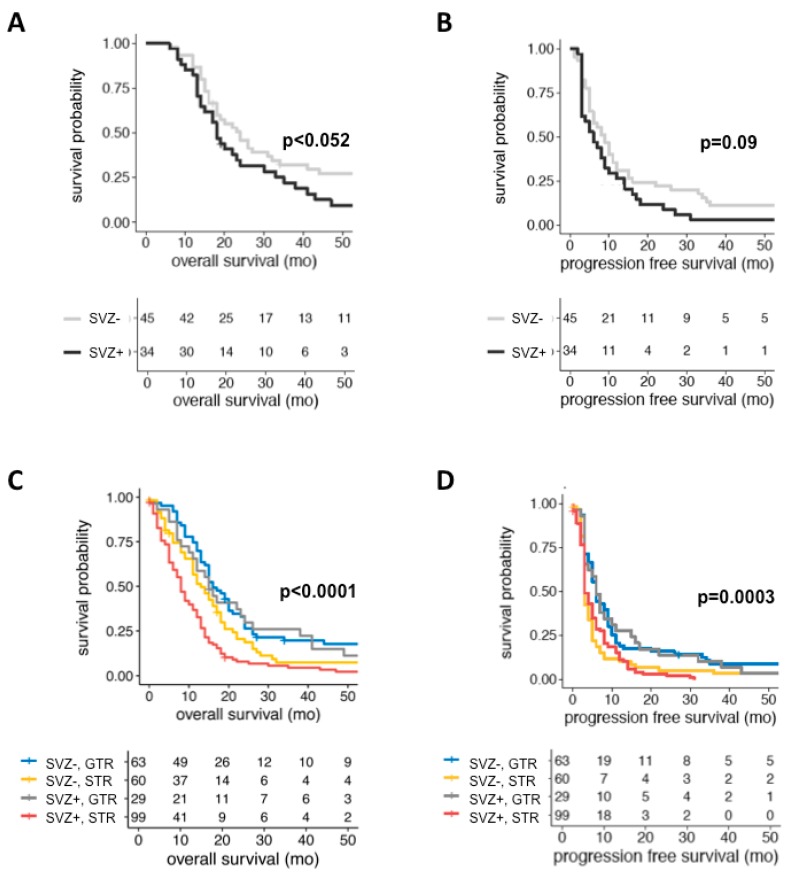
When analyzing IDH1-wt GBM patients with intensified adjuvant treatment (i.e., completion of concomitant radio-chemotherapy and ≥ 3 cycles of TMZ-based chemotherapy) only (*n* = 79), absence of radiographic SVZ involvement conferred a non-significant, but meaningful prolongation of OS (24 vs. 18 months; **A**) and PFS (9 vs. 6 months; **B**) that also compared favorable to the outcomes of the most recent “historic control” derived from a multicenter trial on tumor-treating fields in newly diagnosed GBM [36] (OS = 16 months; PFS = 4 months; data not shown). (**C**,**D**) Kaplan-Meier plots depicting the prognostic significance of SVZ involvement in 285 IDH1-wt GBM patients on OS (**C**) and PFS (**D**) when stratified for EOR. Noteworthy, the negative prognostic impact of SVZ involvement can be, in part, resolved by GTR since OS and PFS were comparable in patients undergoing GTR regardless of SVZ involvement (OS: SVZ+GBM 15.5 months vs. SVZ−GBM = 16.5 months; PFS: SVZ+GBM 6.5 months vs. SVZ−GBM 6 months). A tabular overview of numbers at risk is given below each Kaplan-Meier plot.

Despite its impact on tumor growth and recurrence, cortical involvement was not associated with survival. In contrast, SVZ involvement at first diagnosis and recurrence was predictive of inferior OS and survival after relapse. The negative prognostic impact of SVZ involvement has been described before [16], but our study provides robust survival data from a large cohort that strictly excluded confounding IDH1 mutations [17,18]. Moreover, we applied multivariate survival analysis in which SVZ involvement at first diagnosis was identified as an independent negative prognosticator of OS, and for the first time, SVZ involvement at tumor relapse as an independent negative prognosticator of survival after relapse. Moreover, to exclude a potential bias by group I GBMs that may arise from the cortex, the subcortical white matter or the SVZ, we separately analyzed “pure” SVZ (i.e., group II) and “pure” cortical (i.e., group III) tumors, a comparison that has been disregarded by previous outcome studies. Importantly, SVZ involvement at the first diagnosis remained an independent negative prognosticator of OS. Moreover, SVZ involvement at tumor recurrence conferred significantly shorter survival after relapse (SVZ+ rec: 8 months; SVZ− rec: 13 months; *p* = 0.045), but did not translate into an independent prognostic factor in multivariate analysis. The prognostic significance of SVZ involvement can be best illustrated by comparing our survival data to the most recent “historic control”: In the control arm of a multicenter trial on tumor-treating fields in newly diagnosed GBM [36], patients receiving standard radio-chemotherapy experienced a median PFS and OS of 4 and 16 months, respectively. In our study, analyzing patients with radio-chemotherapy only (*n* = 79), SVZ involvement conferred comparable outcomes (PFS = 6 months; OS = 18 months), while median PFS and OS were meaningfully prolonged to nine and 24 months in SVZ− GBM patients (Figure 4A,B). It has been hypothesized that the poor prognosis associated with SVZ involvement results from an impaired clinical condition and distinct therapeutic challenges encountered with this specific tumor location. Indeed, well-known prognostic factors differed significantly between SVZ+ and SVZ− GBMs since pre-operative KPS, the rate of GTR at the first diagnosis and non-surgical treatment intensity at recurrence were significantly lower in SVZ+ GBMs. Noteworthy, PFS and OS were comparable in patients undergoing GTR regardless of SVZ involvement (SVZ+ GBM: PFS = 6.5 months; OS = 15.5 months; SVZ− GBM: PFS = 6 months; OS = 16.5 months; and Figure 4C,D), underlining the need to strive for maximum safe tumor resection even in tumors involving the lateral ventricles. Nevertheless, all these confounders were included into the multivariate model in which SVZ involvement was confirmed as a robust negative prognostic factor. Admittedly, MGMT promoter methylation status was not considered for multivariate analysis because of missing data in 41% of patients (Table 1) but OS was comparable for patients with (15 months) and without (14 months) methylated MGMT promoter (*p* = 0.255). Therefore, it is reasonable that SVZ involvement does not merely influence survival by the accumulation of negative clinical prognostic factors, but also by its inherent tumor biology. As preliminary evidence, we were recently able to identify molecular markers that were differentially expressed in SVZ+ GBM and conferred a prognostic impact [37].

## 4. Materials and Methods

### 4.1. Patient Cohort

Our institutional database was searched retrospectively for all patients treated for newly diagnosed GBM at the Department of Neurosurgery (University Hospital Heidelberg, Germany) from 2004 to 2011 for whom demographic, treatment-related and outcome data were available and preoperative MRI was accessible on the Picture Archiving and Communication System (PACS). In accordance with the Declaration of Helsinki and the research proposals approved by the Institutional Review Board at Heidelberg Medical Faculty, informed consent was obtained in all cases. (Ethical code: S-005/2003, permission date: 31 March 2003). Histological diagnosis was confirmed by neuropathological review. IDH1 mutation and MGMT promoter methylation status was evaluated as described [2,38,39]. 302 consecutive patients were identified, 285 of those (94.4%) lacking IDH1 mutations. Except for patients with biopsies, EOR was determined for each patient on MRI scans taken within 72 hours post surgery and was valued “complete” (gross total resection = GTR) if no residual contrast enhancement was detected; otherwise, EOR was classified as “subtotal” or “unknown” if no postoperative MRI was available. Adjuvant treatments comprised radiotherapy, concomitant and/or stand-alone TMZ-based chemotherapy as well as treatment within clinical trials. Intensified adjuvant treatment (“Stupp regimen”) was defined as completion of ≥ 3 cycles of TMZ after concomitant radio-chemotherapy. Definition of tumor progression/recurrence was based on the Response Assessment in Neuro-Oncology (RANO) criteria [40] with salvage therapies based on interdisciplinary decision. Salvage treatments comprised re-resection, re-irradiation, cytotoxic (TMZ, nitrosoureas, carboplatin), antiangiogenic (bevacizumab), or targeted therapies. Primary outcome measures were OS, PFS, and survival after relapse. OS was defined as the time from the first histologic diagnosis until death or last follow-up and PFS as the time from first histologic diagnosis to radiographic signs of progression/recurrence or death. Survival after relapse was defined as the time from radiographic signs of progression/recurrence until death. Patients still alive in June 2016 were censored. LTS were defined by an OS of > 36 months [2], while STS lived between six and 10 months after diagnosis.

### 4.2. Radiographic Analysis

For every patient, preoperative and follow-up MRI with standard sequences comprising T1-w, FLAIR and post contrast three-dimensional MPRAGE T1-w images in at least the axial plane with coronal and sagittal reformations were acquired on 1.5 or 3.0 Tesla scanners. As proposed [20], radiographic classification of GBMs according to their vicinity to the SVZ was performed on immediately preoperative CE T1-w MRI by two independent reviewers (CJ, BH). SVZ involvement was valued if the CEL contacted the lining of the ventricle. Group I consisted of tumors contacting the SVZ and infiltrating the cortex, group II of tumors contacting the SVZ only, group III of tumors contacting the cortex only and group IV of tumors residing in the subcortical white matter, neither contacting SVZ nor cortex (Figure 1A). Multifocal disease noncontiguous with the primary CEL was evaluated both on CE T1-w and FLAIR images at 1st diagnosis and at recurrence. At recurrence, distant tumor growth was defined as a new CEL remote from the initial resection cavity. Patients with infratentorial tumor location were precluded from analysis. For comparison of location-dependent outcome, growth and recurrence patterns, patients were further categorized into SVZ+ (groups I, II) versus SVZ− (groups III, IV) GBMs and cortex+ (groups I, III) versus cortex− (groups II, IV) GBMs (Figure 1A). Moreover, group II (involving the SVZ only) and group III (involving the cortex only) tumors were compared separately since this discriminates best between GBMs with and without SVZ involvement.

### 4.3. Statistical Analysis

GraphPad Prism version 6.0c was used for statistical analysis of clinico-pathological patient data as well as growth and recurrence patterns. Survival was analyzed in a Cox proportional hazards model in the *R* package “survival”. Covariate inclusion was defined by stepwise forward selection conducted by the stepAIC algorithm in the *R* package “MASS” (covariates listed in Table S1). Only cases with all covariates available were considered for multivariate analysis. Significance threshold was set at *p* < 0.05.

## 5. Conclusions

In this large-scale analysis of IDH1-wt glioblastomas, tumor location was associated with a distinct growth and recurrence pattern and patient outcome, significantly contributing to the heterogeneous nature of this disease. While the underlying molecular factors still need to be identified, these findings may help to tailor location-dependent treatment strategies and allow for individualized prognostication based on factors easily assessable on pre-operative MRI. SVZ involvement, both at the first diagnosis and tumor relapse, proved to be a robust prognostic factor that should be incorporated in future outcome studies.

## Figures and Tables

**Table 1:** Patient Characteristics of the IDH1-Wildtype Cohort.

Patients; *n* (%)	Group I	Group II	Group III	Group IV	Total	All	SVZ +/−	cortex +/−	II vs. III
	96 (34)	53 (18)	105 (37)	31 (11)	285 (100)	*p*-value			
**Age** (years); median (range)	67 (20–84)	60 (30–81)	61 (36–87)	64 (38–78)	64 (20–87)	0.095	0.404	0.218	0.443
**Sex**; n (male/female)	58/38	34/19	67/38	19/12	178/107	0.953	0.808	1.0	1.0
**KPS pre-op**; median (range)	85 (20–100)	80 (30–100)	90 (40–100)	90 (50–100)	90 (20–100)	**0.002**	**<0.001**	0.092	**0.002**
**Survival Data**									
**Death**; *n* (%)	93 (97)	49 (91)	95 (90)	25 (81)	272 (95)	**0.032**	**0.031**	0.152	0.775
**OS** (months); median (range)	10 (0–69)	8 (0–83)	14 (0–99)	18 (0–68)	12 (0–99)	**<0.001**	**<0.0001**	0.701	**0.018**
**PFS** (months); median (range)	4.5 (0–57)	3 (0–43)	4 (0–90)	5 (0–57)	4 (0–90)	0.197	0.189	0.978	0.271
**Survival after relapse** (months); median (range)	5 (0–49)	6 (0–43)	9 (0–78)	11.5 (0–63)	8 (0–78)	**0.041**	**0.032**	0.286	0.91
**LTS** (> 36 months); *n* (%)	7 (7)	4 (8)	11 (10)	5 (16)	27 (9)	0.478	0.229	0.66	0.775
**STS** (> 6 < 10 months); *n* (%)	21 (22)	11 (20)	16 (15)	4 (13)	52 (18)	0.512	0.167	1.0	0.381
**Molecular Data** (*n* = 285)									
**MGMT meth**; *n* (%) - Yes - No - N/A	**96** (100) 32 (33) 24 (25) 40 (42)	**53** (100) 14 (26) 16 (31) 23 (43)	**105** (100) 20 (19) 42 (40) 43 (41)	**31** (100) 9 (29) 12 (39) 10 (32)	**285** (100) 75 (26) 94 (33) 116 (41)	0.203	**0.045**	0.944	0.394
**Radiographic Characteristics at 1st Diagnosis** (*n* = 285)									
**Multifocal growth**; *n* (%) - CE - FLAIR	**96** (100) 14 (15) 12 (13)	**53** (100) 4 (8) 3 (6)	**105** (100) 18 (17) 19 (18)	**31** (100) 2 (6) 2 (6)	**285** (100) 38 (13) 36 (13)	0.235 0.100	0.602 0.212	0.056 **0.031**	0.146 **0.049**
**Radiographic Characteristics at Recurrence** (*n* = 187)									
**Imaging available**; *n* (%) - Yes - No - Alive & no recurrence	**96** (100) 57 (59) 39 (41) 0 (0)	**53** (100) 28 (53) 25 (47) 0 (0)	**105** (100) 83 (79) 20 (19) 2 (2)	**31** (100) 19 (61) 11 (35) 1 (3)	**285** (100) 187 (66) 95 (33) 3 (1)				
**Location at recurrence**; *n* (%) - Group I (rec) - Group II (rec) - Group III (rec) - Group IV (rec)	**57** (100) 47 (82) 2 (3.5) 4 (7) 4 (7)	**28** (100) 16 (57) 9 (32) 2 (7) 1 (4)	**83** (100) 24 (29) 8 (9.5) 38 (46) 13 (15.5)	**19** (100) 3 (16) 5 (26) 6 (32) 5 (26)	**187** (100) 90 (48) 24 (13) 50 (27) 23 (12)	**<0.0001**	**<0.0001**	**0.0003**	**<0.0001**
**Recurrence pattern**; *n* (%) - Local - Distant - Local & Distant	**57** (100) 41 (72) 3 (5) 13 (23)	**28** (100) 27 (96) 0 (0) 1 (4)	**83** (100) 51 (61) 8 (10) 24 (29)	**19** (100) 19 (100) 0 (0) 0 (0)	**187** (100) 138 (74) 11 (6) 38 (20)	**0.0001**	0.078	**<0.0001**	**0.0002**
**Multifocal growth**; *n* (%) - Multifocal CE (rec) - Multifocal FLAIR (rec)	**57** (100) 18 (32) 7 (12)	**28** (100) 2 (7) 2 (7)	**83** (100) 28 (34) 18 (22)	**19** (100) 2 (11) 2 (11)	**187** (100) 50 (27) 29 (15.5)	**0.013** 0.193	0.409 0.106	**0.001** 0.164	**0.006** 0.096
**Treatment at 1st Diagnosis** (*n* = 285)									
**EOR**; *n* (%) - GTR - Partial - Biopsy - Unknown	**96** (100) 19 (20) 62 (64.5) 1 (1) 14 (14.5)	**53** (100) 10 (19) 30 (57) 6 (11) 7 (13)	**105** (100) 49 (47) 42 (40) 3 (3) 11 (10)	**31** (100) 14 (45) 15 (48) 0 (0) 2 (7)	**285** (100) 92 (32) 149 (52) 10 (4) 34 (12)	**0.0005**	**<0.0001**	0.556	**0.003**
**Adjuvant therapy**; *n* (%) - RT - TMZ concomitant - Stupp - Clinical trial	**96** (100) 77 (80) 46 (48) 22 (23) 29 (30)	**53** (100) 44 (83) 34 (64) 12 (23) 17 (32)	**105** (100) 92 (88) 63 (60) 35 (33) 40 (38)	**31** (100) 27 (87) 19 (61) 10 (32) 8 (26)	**285** (100) 240 (84) 162 (57) 79 (28) 94 (33)	0.289 0.509	0.064 0.451	0.773 0.492	0.199 0.488
**Treatment at Recurrence** (*n* = 187)									
Salvage-therapy; *n* (%) - Treatment received - No treatment received - Lost to follow-up - Alive & no recurrence	**57** (100) 50 (88) 30 17 0	**28** (100) 23 (82) 23 8 0	**83** (100) 73 (88) 19 14 2	**19** (100) 17 (89) 4 9 1	**187** (100) 163 (87) 76 48 3	**0.008**	**0.004**	**0.037**	**0.002**
**Re-resection**; *n* (%) - GTR - Partial - Unknown	**10** (20) 4 (8) 5 (10) 1 (2)	**5** (22) 2 (9) 2 (9) 0	**24** (33) 15 (21) 5 (7) 1 (1)	**6** (35) 3 (18) 1 (6) 2 (12)	**45** (28) 24 (15) 13 (8) 4 (2)	0.24	0.194	0.45	0.538
**Non-surgical therapies**; *n* (%) - 0 (re-resection only) - 1 - 2–5	**57** (100) 2 (4) 34 (68) 14 (28)	**28** (100) 1 (4) 13 (57) 9 (39)	**83** (100) 1 (1) 29 (40) 43 (59)	**19** (100) 0 5 (29) 12 (71)	**187** (100) 4 (2) 81 (50) 78 (48)	**0.013**	**<0.001**	0.789	0.209

KPS. Karnofsky Performance Score; OS: overall survival; PFS: progression-free survival; LTS: long-term survivor; STS: short-term survivor; MGMT: O^6^-methylguanin-DNA-methyltransferase; CE: contrast-enhancing; FLAIR: fluid-attenuated inversion recovery; EOR: extent or resection, GTR: gross total resection; RT: radiotherapy; TMZ: temozolomide; and Stupp: Stupp protocol including >3 cycles TMZ.

**Table 2:** Multivariate Analysis of Overall Survival (*n* = 253), Progression-free Survival (*n* = 253), and Survival after Relapse (*n* = 150) for the Complete IDH1-Wildtype Cohort.

Clinical and Radiographic Factors	*p*-Value	HR	95% CI
**Overall Survival**			
SVZ+ (1st diagnosis)	**0.008** **	1.434	1.099–1.872
Age (above median)	**0.036** *	1.343	1.02–1.77
KPS pre-operative	0.110	0.993	0.985–1.002
EOR: STR (1st diagnosis)	**<0.0001** ***	1.923	1.423–2.599
Intensified Treatment (1st diagnosis)	**<0.0001** ***	0.302	0.221–0.412
Multifocal disease CE (1st diagnosis)	**0.022** *	1.56	1.067–2.280
**Progression-Free Survival**			
SVZ+ (1st diagnosis)	0.529	0.918	0.703–1.199
EOR: STR (1st diagnosis)	**<0.0001** ***	1.811	1.348–2.433
Intensified Treatment (1st diagnosis)	**<0.0001** ***	0.431	0.322–0.579
Multifocal disease FLAIR (1st diagnosis)	**0.013** *	1.614	1.108–2.350
**Survival after Relapse**			
SVZ+ (at relapse)	**0.015** *	1.575	1.092–2.273
Cortex+ (at relapse)	**<0.001** ***	2.069	1.355–3.157
Treatment Intensity (at relapse)	**<0.001** ***	0.768	0.67–0.88
Multifocal disease FLAIR (at relapse)	0.097	1.538	0.925–2.558

HR: Hazard ratio; CI: confidence interval; significance levels: * *p* < 0.05; ** *p* < 0.01; *** *p* < 0.001. SVZ+ (1st diagnosis) [vs. SVZ−]; age (median splitted; 64 years); KPS pre-operative (numeric variable); EOR: STR (subtotal resection) [vs. GTR]; intensified treatment at 1st diagnosis (Stupp protocol including > 3 cycles TMZ); multifocal disease CE; multifocal disease FLAIR; SVZ+ (at relapse) [vs. SVZ−]; cortex+ (at relapse) [vs. cortex−]; treatment intensity at relapse (continuous variable); and multifocal disease FLAIR (at relapse).

**Table 3:** Multivariate Analysis of Overall Survival (*n* = 140), Progression-free Survival (*n* = 140) and Survival after Relapse (*n* = 59) for Group II versus Group III IDH1-Wildtype GBM.

Clinical and Radiographic Factors	*p*-Value	HR	95% CI
**Overall Survival**			
Location group II (1st diagnosis)	**0.007** *	1.725	1.164–2.557
Age (above median)	**<0.0001** ***	2.531	1.690–3.788
KPS pre-operative	0.139	1.009	0.997–1.021
EOR: STR (1st diagnosis)	**<0.001** ***	2.15	1.452–3.184
Intensified Treatment (1st diagnosis)	**<0.0001** ***	0.271	0.179–0.412
**Progression-Free Survival**			
Location group II (1st diagnosis)	0.432	0.848	0.563–1.278
EOR: STR (1st diagnosis)	**0.003** **	1.829	1.233–2.712
Intensified Treatment (1st diagnosis)	**<0.0001** ***	0.406	0.270–0.611
Multifocal disease FLAIR (1stdiagnosis)	0.104	1.519	0.918–2.514
**Survival after Relapse**			
Location group II (at relapse)	0.148	0.623	0.328–1.183
Treatment Intensity (at relapse)	**0.002** **	0.702	0.560–0.879
Multifocal disease FLAIR (at relapse)	**0.005** **	2.966	1.399–6.291

HR: Hazard ratio; CI: confidence interval; significance levels: * *p* < 0.05; ** *p* < 0.01; *** *p* < 0.001. Location group II (1st diagnosis) [vs. group III]; age (median splitted; 64 years); KPS pre-operative (numeric variable); EOR: STR (subtotal resection) [vs. GTR]; intensified treatment at 1st diagnosis (Stupp protocol including > 3 cycles TMZ); multifocal disease FLAIR; Location group II (at relapse) [vs. group III]; treatment intensity at relapse (continuous variable); and multifocal disease FLAIR (at relapse).

## Supplementary Materials

The following are available online at http://www.mdpi.com/2072-6694/11/1/122/s1, Table S1: Covariates included into the multivariate model, Table S2: Univariate Analysis of Survival Endpoints in the Complete IDH1-Wildtype Cohort (*n* = 285), Table S3: Univariate Analysis of Survival Endpoints in Group II and Group III IDH1-Wildtype GBM (*n* = 158).

## Abbreviations

GBM	glioblastoma
WHO	World Health Organization
TMZ	temozolomide
LTS	long-term survivor
NSC	neural stem cell
NPC	neural progenitor cell
SVZ	subventricular zone
PFS	progression-free survival
OS	overall survival
IDH1	isocitrate dehydrogenase 1
CEL	contrast-enhancing lesion
KPS	Karnofsky performance score
GTR	gross total resection
EOR	extent of resection
STS	short-term survivor
MGMT	O^6^-methylguanin-DNA-methyltransferase
CE	contrast-enhancing
T1-w	T1-weighted
FLAIR	fluid-attenuated inversion recovery
RT	radiotherapy
HR	hazard ratio
CI	confidence intervall
PACS	Picture Archiving and Communication System
RANO	Response Assessment in Neuro-Oncology
MPRAGE	magnetization-prepared rapid gradient-echo
