# Supplementary material for: Location-Dependent Patient Outcome and Recurrence Patterns in IDH1-Wildtype Glioblastoma
Source: Cancers (Basel). 2019 Jan 21;11(1):122. doi: 10.3390/cancers11010122 (PMC6356480; doi:10.3390/cancers11010122)
Supplement: Supplementary file 1 [file cancers-11-00122-s001.pdf]

Supplementary Materials: Location-Dependent Patient Outcome and Recurrence Patterns in IDH1-Wildtype Glioblastoma

Christine Jungk, Rolf Warta, Andreas Mock, Sara Friauf, Bettina Hug, David Capper, Amir Abdollahi, Jürgen Debus, Martin Bendszus, Andreas von Deimling, Andreas Unterberg and Christel Herold-Mende

Table S1. Covariates included into the multivariate model.

Complete Cohort			Groups II VS. III		
**Overall Survival**	**PFS**	**Survival after Relapse**	**Overall Survival**	**PFS**	**Survival after Relapse**
		**Covariates at 1st Diagnosis**			
age (median splitted)	age (median splitted)	age (median splitted)	age (median splitted)	age (median splitted)	age (median splitted)
KPS pre-operative	KPS pre-operative		KPS pre-operative	KPS pre-operative	KPS pre-operative
location I vs II vs III vs IV	location I vs II vs III vs IV				
			location II vs III	location II vs III	
multifocal disease CE	multifocal disease CE		multifocal disease CE	multifocal disease CE	
multifocal disease FLAIR	multifocal disease FLAIR		multifocal disease FLAIR	multifocal disease FLAIR	
SVZ involvement	SVZ involvement		SVZ involvement	SVZ involvement	
cortical involvement	cortical involvement		cortical involvement	cortical involvement	
EOR	EOR		EOR	EOR	
intensified treatment	intensified treatment		intensified treatment	intensified treatment	
		**Covariates at Tumor Relapse**			
		location I vs II vs III vs IV			
					location II vs III
		local recurrence			local recurrence
		distant recurrence			distant recurrence
		multifocal disease CE			multifocal disease CE
		multifocal disease FLAIR			multifocal disease FLAIR
		SVZ involvement			SVZ involvement
		cortical involvement			cortical involvement
treatment intensity	treatment intensity	treatment intensity	treatment intensity	treatment intensity	treatment intensity
treatment intensity, non-surgical	treatment intensity, non-surgical		treatment intensity, non-surgical	treatment intensity, non-surgical	

Table S2. Univariate Analysis of Survival Endpoints in the Complete IDH1-Wildtype Cohort (*n* = 285).

**Patients (*n*)**	**OS**	**PFS**	**Survival after Relapse**
	285	285	195
	p-value	*p*-value	*p*-value
**Covariates at 1st Diagnosis**			
Age (median splitted; 64 years)	**<0.0001** ***	**0.016** *	**0.006** **
KPS pre-operative	**0.046** *	0.75	0.34
Location			
- SVZ+ vs. SVZ−	**<0.0001** ***	0.15	**0.023** *
- Cortex+ vs. Cortex-	0.57	0.73	0.31
- Group II vs. III	**0.028** *	0.35	0.8
Multifocal Disease			
- CE	**0.003** **	**0.034** *	**0.005** **
- FLAIR	**0.028** *	**0.002** **	0.21
Extent of Resection			
- GTR vs. STR	**<0.0001** ***	**<0.0001** **	**0.018** *
- GTR vs. NA	**0.006** **	**0.021** *	0.123
Intensified Treatment (Stupp)	**<0.0001** ***	**<0.0001** ***	**<0.0001** ***
**Covariates at Tumor Relapse**			
Location (at relapse)			
- SVZ+ vs. SVZ−	**<0.001** ***	NA	**<0.0001** ***
- Cortex+ vs. Cortex-	**0.001** **	NA	**0.003** **
- Group II vs. III	0.71	NA	0.92
Multifocal Disease (at relapse)			
- CE	0.55	NA	0.11
- FLAIR	0.32	NA	0.31
Recurrence Pattern			
- local	0.29	NA	0.41
- distant	0.78	NA	0.87
Extent of Resection (at relapse)			
- GTR vs. STR	**0.007** **	NA	**0.002** **
- GTR vs. NA	0.579	NA	0.569
Treatment Intensity (at relapse)	**<0.0001** ***	NA	**<0.0001** ***
Non-surgical Treatment Intensity (at relapse)	**<0.001** ***	NA	**<0.0001** ***

OS: overall survival; PFS: progression-free survival; KPS: Karnofsky Performance Score; CE: contrast enhancement; FLAIR: fluid-attenuated inversion recovery; GTR: gross total resection; STR: subtotal resection; NA: EOR not available; ns: not significant; NA: not applicable, significance levels: * $p < 0.05$; ** $p < 0.01$; *** $p < 0.001$.

Table S3. Univariate Analysis of Survival Endpoints in Group II and Group III IDH1-Wildtype GBM (n = 158).

**Patients (*n*)**	**OS**	**PFS**	**Survival after Relapse**
	143		60
	p-value	*p*-value	*p*-value
**Covariates AT 1st Diagnosis**			
Age (median splitted; 64 years)	**<0.0001** ***	**0.002** **	**0.008** **
KPS pre-operative	0.48	0.56	**0.033** *
Location			
- Group II vs. III	**0.028** *	0.35	0.8
Multifocal Disease			
- CE	**0.023** *	0.13	0.068
- FLAIR	**0.023** *	**0.003** **	0.25
Extent of Resection			
- GTR vs. STR	**<0.0001** ***	**<0.0001** ***	0.25
- GTR vs. NA	**0.035** *	0.015	0.44
Intensified Treatment (Stupp)	**<0.0001** ***	**<0.0001** ***	**0.001** **
**Covariates at Tumor Relapse**			
Location (at relapse)			
- SVZ+ vs. SVZ−	0.074	NA	**0.045** *
- Cortex+ vs. Cortex-	0.082	NA	0.063
- Group II vs. III	0.97	NA	0.99
Multifocal Disease (at relapse)			
- CE	0.36	NA	0.12
- FLAIR	0.083	NA	**0.031** *
Recurrence Pattern			
- local	0.92	NA	0.78
- distant	0.67	NA	0.46
Extent of Resection (at relapse)			
- GTR vs. STR	**0.014** *	NA	**<0.001** ***
- GTR vs. NA	0.759	NA	0.628
Treatment Intensity (at relapse)	**0.015** *	NA	**0.003** **
Non-surgical Treatment Intensity (at relapse)	**0.019** *	NA	**0.007** **

OS: overall survival; PFS: progression-free survival; KPS: Karnofsky Performance Score; CE: contrast enhancement; FLAIR: fluid-attenuated inversion recovery; GTR: gross total resection; STR: subtotal resection; NA: EOR not available; ns: not significant; NA: not applicable, significance levels: * $p < 0.05$; ** $p < 0.01$; *** $p < 0.001$.

© 2019 by the authors. Licensee MDPI, Basel, Switzerland. This article is an open access article distributed under the terms and conditions of the Creative Commons Attribution (CC BY) license (http://creativecommons.org/licenses/by/4.0/).
